# Heterogeneous SARS-CoV-2-Neutralizing Activities After Infection and Vaccination

**DOI:** 10.3389/fimmu.2022.888794

**Published:** 2022-05-30

**Authors:** Marianne Graninger, Jeremy V. Camp, Stephan W. Aberle, Marianna T. Traugott, Wolfgang Hoepler, Elisabeth Puchhammer-Stöckl, Lukas Weseslindtner, Alexander Zoufaly, Judith H. Aberle, Karin Stiasny

**Affiliations:** ^1^ Center for Virology, Medical University of Vienna, Vienna, Austria; ^2^ Department of Medicine IV, Clinic Favoriten, Vienna, Austria

**Keywords:** SARS-CoV-2, COVID-19, SARS-CoV-2-specific antibody responses, SARS-CoV-2-neutralizing antibodies, variants of concern

## Abstract

**Background:**

Severe acute respiratory syndrome coronavirus 2 (SARS-CoV-2) variants of concern (VOCs) with different resistance levels to existing immunity have recently emerged. Antibodies that recognize the SARS-CoV-2 spike (S) protein and exhibit neutralizing activities are considered the best correlate of protection and an understanding of humoral immunity is crucial for controlling the pandemic. We thus analyzed such antibodies in individuals recovered from infection in 2020 as well as vaccinees after two doses of an mRNA vaccine.

**Methods:**

Neutralizing antibody responses against three SARS-CoV-2 variants (D614G, VOCs Beta and Delta) were determined in serum samples from 54 infected individuals (24 non-hospitalized, 30 hospitalized) and 34 vaccinees shortly after symptom onset or second vaccination, respectively, as well as six months later. In addition, the effect of the S sequence of the infecting strain on neutralization was studied.

**Results:**

Non-hospitalized patients had the lowest neutralization titers against all variants, while those of hospitalized patients equaled or exceeded those of vaccinees. Neutralizing activity was lower against the two VOCs and declined significantly in all cohorts after six months. This decrease was more pronounced in hospitalized and vaccinated individuals than in non-hospitalized patients. Of note, the specific neutralizing activity (NT titer/ELISA value ratio) was higher in the infected cohorts than in vaccinees and did not differ between non-hospitalized and hospitalized patients. Patients infected with viral strains carrying mutations in the N-terminal domain of the spike protein were impaired in Beta VOC neutralization.

**Conclusions:**

Specific neutralizing activities were higher in infected than in vaccinated individuals, and no difference in the quality of these antibodies was observed between hospitalized and non-hospitalized patients, despite significantly lower titers in the latter group. Additionally, antibody responses of infected individuals showed greater heterogeneity than those of vaccinees, which was associated with mutations in the spike protein of the infecting strain. Overall, our findings yielded novel insights into SARS-CoV-2-specific neutralizing antibodies, evolving differently after virus infection and COVID-19 vaccination, which is an important issue to consider in ongoing vaccine strategy improvements.

## Introduction

Since its emergence, severe acute respiratory syndrome coronavirus 2 (SARS-CoV-2) has evolved into multiple variants, some with mutations that affect their biology, spread and transmissibility. These include the variants of concern (VOCs) Alpha, Beta, Gamma, Delta and the most recently emerged Omicron, which exhibit different levels of immune escape^1^.

Neutralizing antibodies are predictive for protection against disease (coronavirus disease 2019, COVID-19) (1–3), but the precise definition of such correlates has remained elusive so far, requiring a better understanding of the different immune responses induced by infection and/or vaccination. Severe COVID-19 and mRNA vaccinations were shown to induce high amounts of antibodies, whereas lower titers were observed after asymptomatic or mild infections (4–7). Waning of virus-specific IgG and neutralizing antibodies has been observed in all instances, and its influence on long-term protection, in particular with respect to VOCs, is a matter of intensive research (8). In our study, we therefore investigated antibody responses in groups of individuals after SARS-CoV-2 infection, either hospitalized or non-hospitalized, and vaccination using samples collected early (~ three weeks) and late (~six months) after antigen exposure.

The majority of the neutralizing activity after infection is attributable to antibodies binding to the receptor-binding domain (RBD) of the surface spike glycoprotein (S) (9–11). The spike protein is not only responsible for receptor binding, but also mediates membrane fusion during host cell entry [reviewed in (12)]. Additionally, potent neutralizing antibodies have been described to target the N-terminal domain (NTD) of the spike, which, like the RBD, is part of the S1 domain of S (13–15). An RBD-specific neutralization dominance was also observed following vaccination with mRNA vaccines (4, 16). Both licensed mRNA vaccines encode a membrane-anchored SARS-CoV-2 spike protein with the sequence of the strain originally isolated in Wuhan and two stabilizing proline mutations in the membrane-anchored S2 part (17, 18).

As neutralizing antibodies play a critical role in preventing virus infections, we investigated the humoral immunity in hospitalized and non-hospitalized patients as well as mRNA vaccinees early after disease onset or the two-dose vaccination, respectively, and six months later, including neutralization of three SARS-CoV-2 variants (an ancestral D614G strain and the two VOCs Beta and Delta). We found that the specific neutralizing activity (defined as the ratio of neutralizing antibodies to SARS-CoV-2 S-specific IgG) is higher in infected than in vaccinated people regardless of severity of disease and the sampling time point. In addition, the range of these ratios was larger after infection than after vaccination. Analysis of spike sequences in patient swabs indicated that the observed heterogeneity was associated with additional mutations in the NTD of the infecting virus strain.

## Methods

### Human Samples and Ethical Statement

All patients (non-hospitalized and hospitalized) had been diagnosed with SARS-CoV-2 infection by PCR testing from nasal swabs or respiratory secretions between March and November 2020, excluding the possibility of infection with a VOC. All patients gave written informed consent before being included in the study.

Serum samples after the second dose of the BNT162b2 mRNA vaccine from healthcare workers were sent to the diagnostic laboratory of the Center for Virology for antibody titer determinations, and anonymized leftover samples were used for our analyses. SARS-CoV-2 infections were ruled out by regular PCR testing of the vaccinees.

Serum samples were collected approximately three weeks after infection/vaccination (“early” samples, median 19 days, range 9-41 days) as well as approximately six months later (“late” samples, median 176 days, range 107-429 days). Median time to sample collection after symptom onset or second vaccination was similar in all cohorts, as shown in **Table 1**.

**Table 1:** Description of study cohorts.

	Total	Non-hospitalized	Hospitalized	BNT162b2 vaccinated
N	88	24	30*	34
		Symptomatic (% of N)	ICU (% of N)	
		24 (100)	13 (43)	
**Median age in years [range]**	37 [19-89]	25 [20-35]	51 [19-89]	45 [20-58]
**Male sex (%)**	43 (49)	15 (63)	23 (77)	5 (15)
**“Early” samples in dpo/dpv [range]**	19 [9-41]	25 [14-41]	18 [9-25]	17 [10-28]
**“Late” samples in dpo/dpv [range]**	176 [107-429]	184 [170-219]	235 [169-429^†^]	162 [107-183]
**Sequenced (% of N)**	25 (28)	4 (17)	19 (63)	n/a

dpo, days post symptom onset; dpv, days post 2nd vaccination; n/a, not applicable; ICU, intensive care unit.

*For seven patients, serum samples were available only at the early time point. These samples were not included in longitudinal analyses.

^†^Late samples of two patients were collected at days 352 and 429 post symptom onset, respectively. All other “late” samples were collected between month 6 and month 9 post symptom onset (range: dpo 169–288).

#### Ethics Statement

The analyses were approved by the local ethics committee of the Medical University of Vienna (EK-No. 1291/2021 and EK-No. 1926/2020).

### Viruses

The D614G, Beta and Delta SARS-CoV-2 strains were isolated from nasopharyngeal swabs from COVID-19 patients. Vero E6 cells (ECACC 85020206) were infected and incubated at 37°C until a cytopathic effect occurred. Cell culture supernatant was harvested and the presence of SARS-CoV-2 was confirmed by PCR. The virus isolates were then passaged two more times in Vero E6 cells. All isolates were controlled to be free of other respiratory viruses by PCR as described (19), and were tested negative for mycoplasma contamination by the MycoAlertTM Mycoplasma Detection Kit (Lonza Group Ltd, Basel, Switzerland). The sequences were determined by next generation sequencing and uploaded to the GISAID database. GISAID accession numbers: D614G variant, EPI_ISL_438123/hCoV-19/Austria/CeMM0360/2020 (19); Beta VOC, EPI_ISL_4236051 (mutations D80A, D215G, L242‐, A243‐, L244‐, K417N, E484K, N501Y, D614G, A701V); Delta VOC, EPI_ISL_4172121 (mutations T19R, G142D, E156del/F157, R158G, L452R, T478K, D614G, P681R, R682W, D950). The cell culture-adaptation R682W acquired in the Delta VOC has been shown not to influence virus neutralization (5).

#### Sequencing

Whole genome sequencing of SARS-CoV-2 was performed using a tiled amplicon approach with the ARTICv3 primer panel. Amplicon pools were fragmented and multiplexed with dual index barcodes (NexteraXT, Illumina, Inc.). The cleaned, indexed libraries were pooled in equimolar ratios and sequenced on an Illumina MiSeq, using V2 chemistry in paired reads of 150 bp in each direction. Sequencing reads were demultiplexed, quality trimmed, and aligned to the Wuhan-Hu-1 reference sequence (GenBank accession number: NC_045512.2) by the BWA-MEM software package (20). Primer sequences were masked using iVar package, and the consensus FASTA file was generated from the BAM file using samtools and mpileup a majority vote to exclude minor variants (21, 22). All sequenced strains had a minimum coverage of 200X.

### RBD-Specific IgG ELISA

Spike-specific IgG antibodies were quantified in binding antibody units (BAU)/ml using the WANTAI SARS-CoV-2 IgG ELISA kit (Beijing Wantai Biological Pharmacy Ent.). The test is based on the RBD of the original strain isolated in Wuhan and was performed according to the manufacturer’s instructions. Samples were serially diluted until the endpoint in all instances.

### Neutralization Assays

The live virus neutralization tests (NT) were performed as previously described (19). Briefly, two-fold serial dilutions of heat-inactivated serum or plasma samples were incubated with 50–100 TCID_50_ SARS-CoV-2 (D614G strain, Beta or Delta VOCs) for one hour at 37°C before the mixture was added to Vero E6 cell monolayers (starting dilution of samples 1:10). After three days, NT titers were expressed as the reciprocal of the serum dilution required for protection against virus-induced cytopathic effect. NT titers ≥10 were considered positive.

### Statistical Analysis

Statistical analysis was performed with GraphPad Prism (Version 9.2.0). For longitudinal analyses, only samples from patients available at both the early and late time points were included. Seven additional early samples were included in the spike sequence analyses (**Figure 6**).

RBD-ELISA BAU/ml and NT titers were compared with the Kruskal-Wallis test (3 groups, Dunn’s multiple comparisons *post hoc* test) or Mann-Whitney test (2 groups). In the case of NT titers < 10, a value of 5 was used for these analyses. Fold reductions of antibody levels over time were calculated for each patient individually using the following formulas:


$$fold\;reduction\;of\;anti\;body\;level\;=\frac{BAU\;per\;ml,\;early\;sample}{BAU\;per\;ml,\;late\;sample}$$

or


$$\frac{NT\;titer,\;early\;sample}{NT\;titer,\;late\;sample}$$

This analysis included only patients with a positive test result in all assays at both time points.

Specific neutralization (NT/ELISA ratios) was calculated for samples that were positive in both assays and log-transformed for ANOVA (three groups, Tukey’s multiple comparisons *post hoc* test)or a paired t-test (two groups). The ranges of NT/ELISA ratios were expressed as maximum-to-minimum factors, which were calculated by dividing the maximum ratio through the minimum ratio for each cohort, time point and virus variant, respectively. Spearman’s rho was applied to evaluate the correlation between the test results (ELISA and NT), to assess the correlation between NT titers against different variants, and the correlation between ELISA, NT or NT/ELISA ratios and the time point of sample collection (days post onset/2^nd^ vaccination), respectively. The coefficient of determination (R^2^) was used to assess variability.

## Results

### Study Cohorts

The study population consisted of 54 patients infected between March and November 2020 (24 non-hospitalized and 30 hospitalized) and 34 individuals after a two-dose mRNA vaccination (BNT162b2).

SARS-CoV-2 infection was diagnosed by RT-PCR from nasal swabs or respiratory secretions. Non-hospitalized patients showed a mild to moderate course of the disease. Thirteen of 30 (43%) hospitalized patients were treated in intensive care units (**Table 1**).

Serum samples were taken at two time points. The first sample was collected approximately three weeks post symptom onset or the second vaccine dose (“early” samples, median 19 days, range 9-41 days). Follow-up samples were collected approximately six months later (“late” samples, median 176 days, range 107-429 days). As shown in **Table 1**, the sampling time points were similar in all cohorts. Infected individuals were predominantly male, and hospitalized patients were older than non-hospitalized patients.

### IgG Antibody Responses

**Figure 1:**
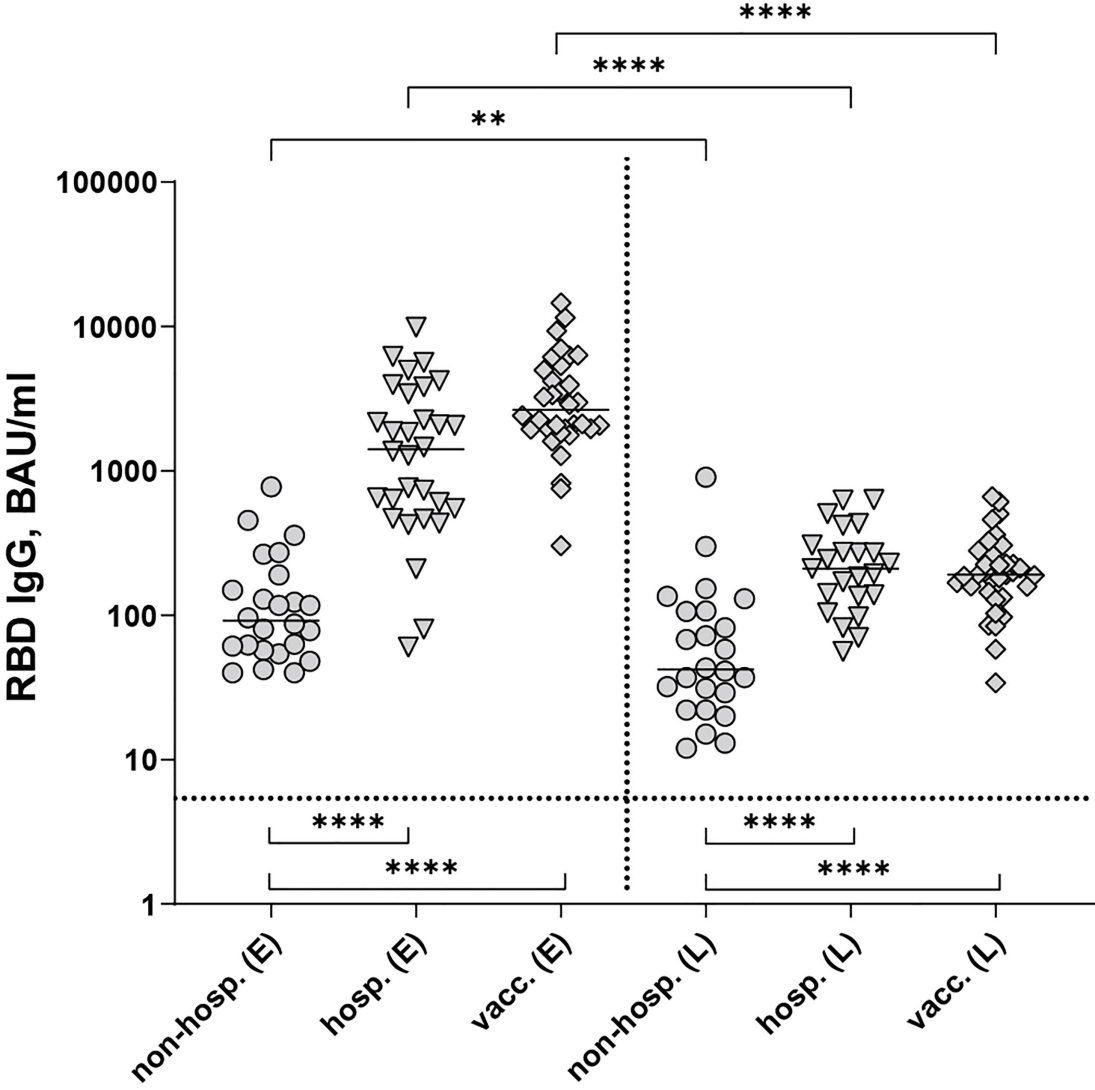
SARS-CoV-2 RBD-specific IgG antibodies after infection (non-hospitalized and hospitalized patients) and two doses of the mRNA vaccine BNT162b2. Early samples (E) were taken approximately three weeks after disease onset or the second vaccine dose; late samples (L) approximately six months after disease onset or the second vaccine dose. Bars indicate the median, the dotted horizontal line the cut-off of the assay. Statistical analysis was performed with the Kruskal-Wallis test and Dunn’s multiple comparisons *post hoc* test (comparison of cohorts, bottom brackets) or Mann-Whitney test (comparison within groups, top brackets). Asterisks indicate statistical significance: (**) = P ≤ 0.01, (****) = P ≤ 0.0001. non-hosp., non-hospitalized patients; hosp., hospitalized patients; vacc., vaccinees.

Non-hospitalized patients yielded the lowest RBD-specific IgG antibody concentrations at both time points (*P <* 0.0001), whereas hospitalized and vaccinated individuals developed similar amounts of antibodies (*P* > 0.05, **Figure 1**). Antibody concentrations declined significantly in all cohorts within six months post disease onset or after the second vaccination (**Figure 1**), with the strongest waning occurring in vaccinated individuals (**Supplementary Table 1**).

### Neutralizing Antibody Responses

**Figure 2:**
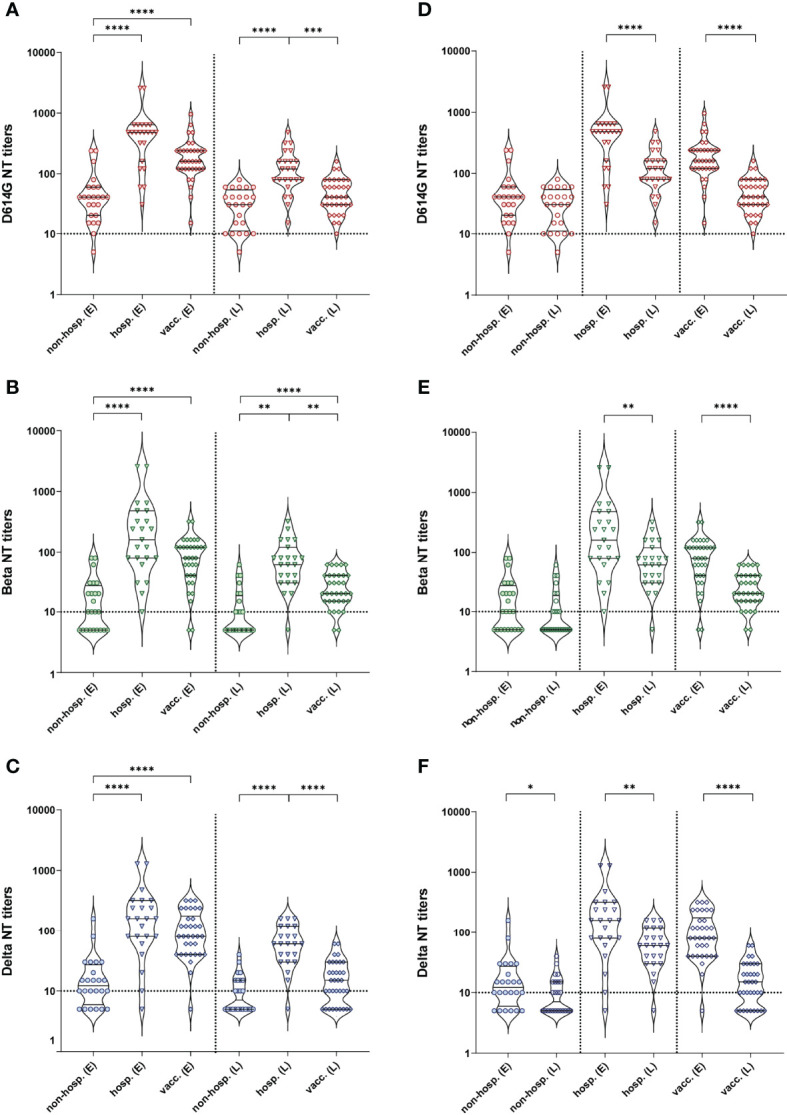
Neutralizing antibody titers against an ancestral D614G virus strain and the two VOCs Beta and Delta after infection (non-hospitalized and hospitalized patients) and two doses of the mRNA vaccine BNT162b2. **(A–C)** Comparison between cohorts. **(D–F)** Graphs represent the same data as in **(A–C)**, but regrouped according to time points of sample collection. **(A, D)** D614G virus NT, **(B, E)** Beta VOC NT, **(C, F)** Delta VOC NT. Early samples (E) were taken approximately three weeks after disease onset or the second vaccine dose; late samples (L) approximately six months after disease onset or the second vaccine dose. Bars within the violin plots indicate the median with interquartile range, the dotted horizontal line the cut-off of the assay. Statistical analysis was performed by Kruskal-Wallis test and Dunn’s multiple comparisons *post hoc* test **(A–C)** or Mann-Whitney test **(D–F)**. Asterisks indicate statistical significance: (*) = P ≤ 0.05, (**) = P ≤ 0.01, (***) = P ≤ 0.001, (****) = P ≤ 0.0001. non-hosp., non-hospitalized patients; hosp., hospitalized patients; vacc., vaccinees.

To analyze functional activities of antibodies, we performed NTs with all samples using an isolate from the early pandemic (D614G virus) and the two VOCs Beta (B.1.351) and Delta (B.1.617.2). In line with our ELISA results, non-hospitalized patients showed significantly lower NT titers against all virus strains than hospitalized and vaccinated individuals at both time points, whereas hospitalized individuals developed the highest titers against all variants at each time point (**Figures 2A–C**). At the early time point, no significant difference in NT titers was observed between hospitalized and vaccinated individuals, while six months later hospitalized patients showed significantly higher NT titers than vaccinees (**Figures 2A–C**). For all cohorts, NT titers were highest against the D614G strain at both time points (**Supplementary Figure 1**), and no significant differences were observed between the Beta and Delta VOCs.

A decline in NT titers over time was observed in all cohorts for the three virus strains (**Figures 2D–F**), which was significant for the vaccinated and hospitalized cohorts. In the case of non-hospitalized individuals, a significant decrease was only detected for the Delta variant (**Figure 2F**; **Supplementary Table 1**).

**Figure 3:**
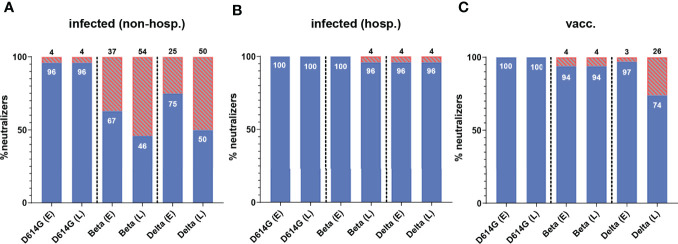
Percentage of individuals with neutralizing activity against an ancestral D614G virus strain and the two VOCs Beta and Delta in **(A)** non-hospitalized and **(B)** hospitalized patients and **(C)** individuals after two doses of the mRNA vaccine BNT162b2. Early samples (E) were taken approximately three weeks after disease onset or the second vaccine dose; late samples (L) approximately six months after disease onset or the second vaccine dose. The percentage of serum samples with a positive (blue) and negative (red, lined) result for both time points are shown. Numbers in/on bars indicate the percentage of NT-positive and NT-negative individuals, respectively. non-hosp, non-hospitalized patients; hosp, hospitalized patients; vacc, vaccinees.

At both time points, all hospitalized and vaccinated individuals had neutralizing antibodies against the D614G strain, and only one non-hospitalized patient did not develop such antibodies (**Figures 2**, **3**). In contrast, a substantial number of non-hospitalized patients were negative against the Beta and Delta VOCs at the early time point (37% and 25%, respectively), with a further decline during the six-month period (54% and 50%, respectively; **Figure 3A**). In terms of the proportion of samples with detectable neutralizing activity against the Beta and Delta VOCs, the highest percentages of NT-positive samples were from hospitalized patients (**Figure 3B**), with only a few individuals negative in the Beta and Delta VOC NTs at both time points (**Figure 3B**). A similar result was obtained for vaccinees in the NT with the Beta variant (**Figure 3C**), but while only 3% were negative in the Delta VOC NT at the early time point, this proportion increased to 26% over the following six months (**Figure 3C**).

### Individual Variation of Antibody Responses

**Figure 4:**
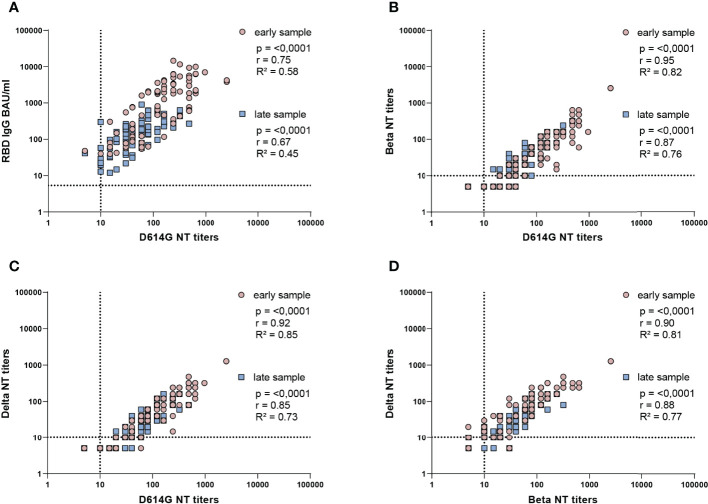
Correlation analysis of ELISA concentrations and NT titers of all samples. Spearman correlation coefficients (r) and coefficients of determination (R^2^) are indicated. **(A)** IgG in BAU/ml and D614G virus NT titers, **(B)** D614G virus and Beta VOC NT titers, **(C)** D614G virus and Delta VOC NT titers, **(D)** Beta VOC and Delta VOC NT titers. All samples from infected and vaccinated individuals for which early and late samples were available are included. Early samples were taken approximately three weeks after disease onset or the second vaccine dose; late samples approximately six months after disease onset or the second vaccine dose. The dotted lines indicate the cut-offs of the specific assays.

To assess the relationships between ELISA-binding and D614G-neutralizing antibodies (**Figure 4A**) as well as NTs with different SARS-CoV-2 strains (**Figures 4B–D**), we analyzed the respective assay values (BAU/ml, NT titers) by Spearman correlation tests. Overall, significant positive correlations were observed in all instances (p = < 0.0001, r = 0.67-0.95), but a certain variability was observed, especially in the ELISA-NT analysis with the lowest R² values (R² = 0.58 and 0.45, **Figure 4A**).

**Figure 5:**
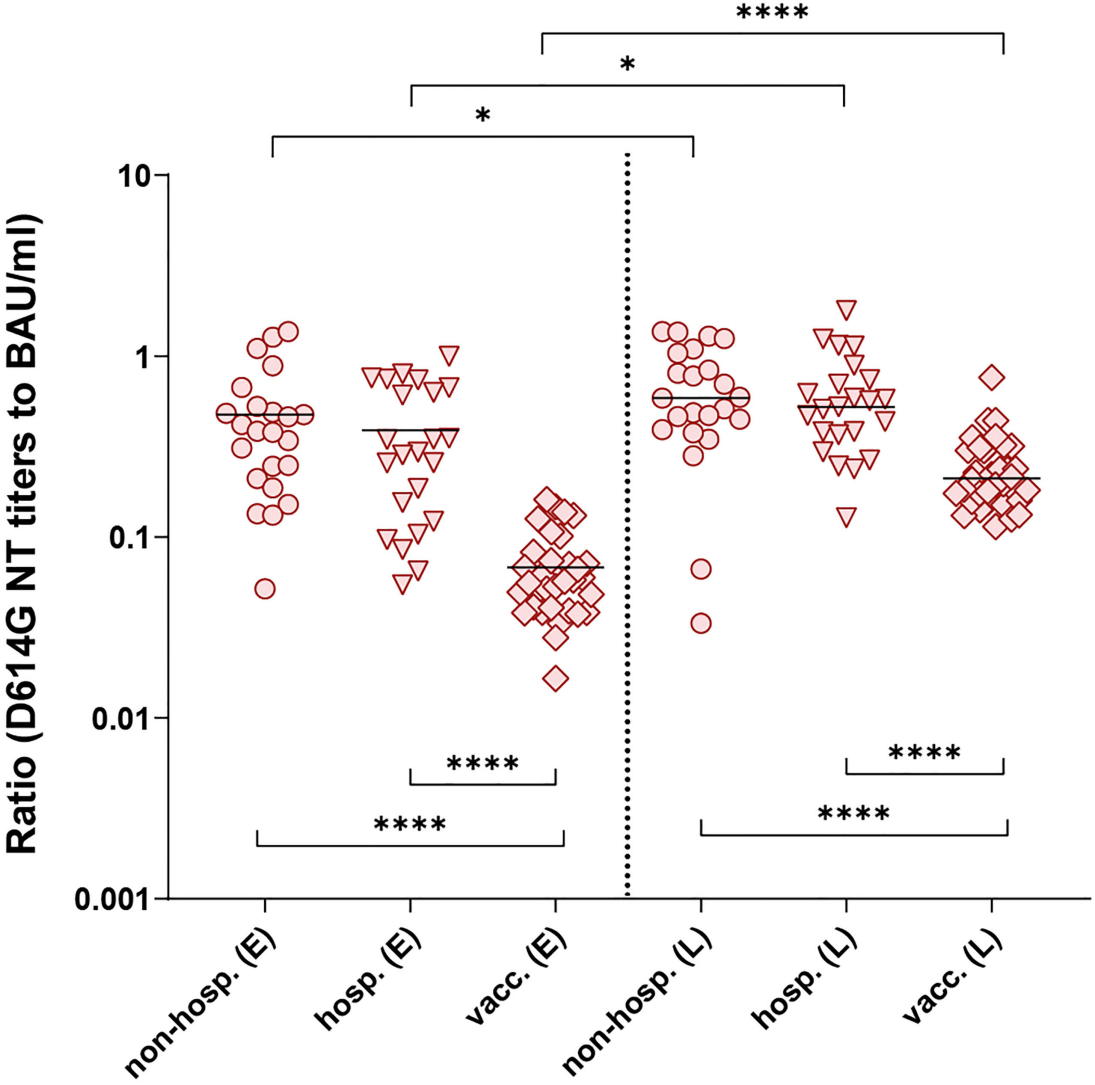
Ratios of D614G virus NT titers and ELISA concentrations of individual serum samples after infection (non-hospitalized and hospitalized patients) and two doses of the mRNA vaccine BNT162b2. Only NT- and ELISA-positive samples were included. Early samples (E) were taken approximately three weeks after disease onset or the second vaccine dose; late samples (L) approximately six months after disease onset or the second vaccine dose. Bars indicate the mean. Statistical analysis was performed with ANOVA and Tukey’s multiple comparison’s *post hoc* test (comparison of cohorts, bottom brackets) and paired t-test (comparison within groups, top brackets). Asterisks indicate statistical significance: (*) = P ≤ 0.05, (****) = P ≤ 0.0001. non-hosp., non-hospitalized patients; hosp., hospitalized patients; vacc., vaccinees.

To investigate the specific NT activities of each individual in detail, we compared the ratios between neutralizing and ELISA-binding antibodies for the three groups (specific NT activities) (**Figure 5**). NT/ELISA ratios were only calculated for samples with a positive result in both assays, which was the case for most samples tested in the D614G NT (only one non-hospitalized patient with a negative NT had to be excluded, see **Figures 2** and **3**). In contrast, a number of samples showed no neutralization of the Beta or Delta VOCs (**Figures 2**, **3**), especially in the non-hospitalized cohort.

We found no differences in the D614G NT/ELISA ratios between non-hospitalized and hospitalized individuals at both time points (**Figure 5**), but the ratios after infection were significantly higher than those obtained from vaccinated individuals, indicating either the presence of more strongly neutralizing antibodies and/or a higher proportion of such antibodies. In all cohorts, an improvement of these ratios was observed up to the late time point. Similar results were found with the ratios calculated for the Beta and Delta VOCs (**Supplementary Figure 2**). In all instances, the maximum-to-minimum factors of the NT/ELISA ratios were higher after infection than after vaccination (**Supplementary Table 2**), revealing a greater variability within the infected groups.

To analyze the antibody responses in relation to the time points of sample collection, we performed correlation analyses combining the WT-specific antibody levels as well as the NT/ELISA ratios from all cohorts (**Supplementary Figure 3**). When we plotted the RBD IgG ELISA values and D614G NT titers against the early sampling time points, we saw a moderate negative correlation (Spearman r = -0.4 and -0.35, respectively) (**Supplementary Figures 3A, C**). No such effect was seen for the early NT/ELISA ratios (Spearman r = 0.23) (**Supplementary Figure 3E**) or with any of these parameters at late time points (**Supplementary Figures 3B, D, F**). In the latter instances, we obtained only a very weak or no correlation (r = 0.06 – 0.26). The individual variation, however, was very high for all combinations tested (R² ≤ 0.1).

### Influence of the Spike Sequence on Antibody Responses

As we observed more heterogeneous functional antibody responses in infected than in vaccinated individuals (**Figure 5**; **Supplementary Figure 2**; **Supplementary Table 2**), which could not be explained by the sampling time point (**Supplementary Figures 3E, F**), we hypothesized that mutations in the S sequence of the infecting strain might influence virus neutralization in individual patients. Leftover material from swabs initially used for the diagnosis of SARS-CoV-2 infection was available from 22 patients (**Table 2**) and was used for sequencing the spike gene of the respective infecting strain. Fifteen of these patients showed neutralizing antibodies against all variants at both time points. Of the remaining seven patients, only early blood samples were available, which were also NT positive against all variants.

**Table 2:** Location of spike mutations identified in a subset of samples from infected individuals.

Cohorts	n	sequenced (% of n)	Mutations found in spike sequences of infecting strains (individuals, n)							
			No mutation	D614G	D614G+RBD	D614G+NTD	D614G+RBD+NTD	D614G+RBD+S2	D614G+NTD+S2	D614G+S1
**Total**	54	22 (41)	1	10	1	5	1	1	2	1
**Non-Hospitalized**	24	2 (8)	0	1	1	0	0	0	0	0
**Hospitalized**	30*	20 (67)	1	9	0	5	1	1	2	1

RBD, receptor-binding domain; NTD, N-terminal domain; S1, S1 subunit; S2, S2 subunit.

*samples of seven patients only available at early time point.

**Figure 6:**
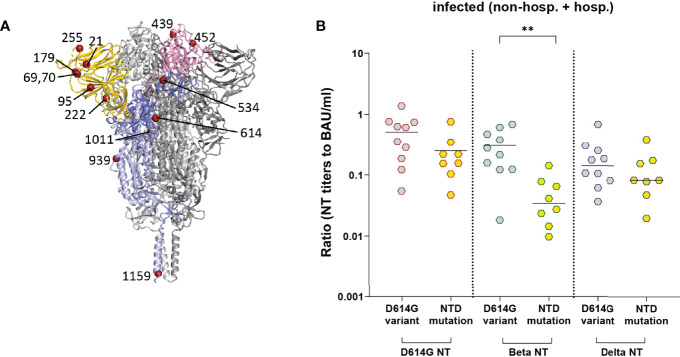
NT/ELISA ratios in infected individuals (non-hospitalized and hospitalized patients) with known S sequence of the infecting strain. **(A)** Ribbon representation of a G614 S protein (PDB: 7KRQ (23)). Two protomers are shown in grey; one is colored according to S domains (NTD, gold; RBD, pink; S1, medium blue; S2, light blue). The mutations identified in the infecting strains (**Table 3**) are shown as dark red spheres. **(B)** NT/ELISA ratios in early samples of patients infected with the D614G mutation versus D614G plus additional NTD mutations. Early samples were taken approximately three weeks after disease onset. The bars indicate the mean. Statistical analysis was performed with unpaired t-tests. Asterisks indicate statistical significance: (**) = P ≤ 0.01. non-hosp., non-hospitalized patients; hosp., hospitalized patients.

One patient was infected with the D614 strain (originally isolated in Wuhan), ten samples had the D614G mutation only, and samples of eleven patients carried one or two additional substitutions in different regions of S (**Figure 6A**) in addition to D614G (**Tables 2**, **3**) as compared to the reference sequence (see Methods). As shown in **Figure 6A**, most mutations in the study cohort were located in the NTD. Therefore, we compared the NT/ELISA ratios from the D614G-infected patients (n = 10) with those that had additional mutations in the NTD (n = 8) (**Figure 6B**). Since we had only early samples for five of these patients, an analysis of the late time point was not possible. Patients previously infected with NTD-mutated strains showed a slight reduction in the D614G- and Delta-NT/ELISA ratios compared to patients infected with viruses carrying only the D614G mutation (**Figure 6B**), which was significant in the case of the Beta VOC, suggesting a possible association between S sequence and variance in neutralizing activities.

**Table 3:** Specific S mutations identified in infected individuals.

Site of mutation	Amino acid substitutions (in addition to D614G)	Cohort (individuals, n)
**No mutation**	–	non-hosp (1).
**D614G**	–	non-hosp (1)., hosp (9).
**D614G+RBD**	L452R	non-hosp (1).
**D614G+NTD**	S255F	hosp (3).
	R21I	hosp (1).
	A222V	hosp (1).
**D614G+RBD+NTD**	N439K+Del69/70	hosp (1).
**D614G+NTD+S2**	T95I + H1159Y	hosp (1).
	L179F + S939F	hosp (1).
**D614G+RBD+S2**	S477N +Q1011fs	hosp (1).
**D614G+S1**	V534F	hosp (1).

RBD, receptor-binding domain; NTD, N-terminal domain; S1, S1 subunit; S2, S2 subunit.

## Discussion

In this study, we provide a comprehensive analysis of the variability of antibody responses in longitudinal cohorts after early-pandemic infection with SARS-CoV-2 and vaccination with an mRNA COVID-19 vaccine. The data presented demonstrate not only substantial differences in the magnitude as well as the functional activity of the induced antibodies, but also a greater heterogeneity of responses in infected than in vaccinated individuals.

Non-hospitalized and hospitalized patients developed antibodies with similar functional activities (NT/ELISA ratios in **Figure 5**), despite significantly lower antibody titers in non-hospitalized (low responder) versus hospitalized (high responder) individuals (**Figure 2**). On the other hand, vaccinees had similar NT titers as hospitalized patients at the early time point and fell between the high- and low responder patients six months later (**Figure 2**), but the NT/ELISA ratios of vaccine-induced antibodies were significantly lower at both time points (**Figure 5**), suggesting the presence of more non-neutralizing antibodies. Such differences between infection- and vaccine-induced antibodies were also described by others and are consistent with studies reporting rather low proportions of plasmablasts generating neutralizing antibodies in vaccinated individuals (4, 16, 24). The reasons for these discrepancies are unclear, but they could be due to differences in the presentation of the S protein to the immune system. In virus particles, S is associated with other structural proteins, whereas the mRNA for the synthesis of S in the vaccine lacks these components, possibly leading to modulations of glycosylation patterns and/or S stability [reviewed in (12)]. S in the vaccine, based on the original strain isolated in Wuhan (17, 25), has two proline mutations in the S2 domain to avoid the adoption of the inactive post-fusion state [reviewed in (12)], which can slightly alter the conformational landscape of the pre-fusion S protein and might have contributed to the differences observed in our study (26, 27).

In infected and vaccinated individuals, waning of antibodies occurred during the intervening six-month period, but all SARS-CoV-2 IgG values (RBD of the original Wuhan strain as ELISA antigen) and most NT titers against the D614G virus were still positive at this time point (**Figures 1**–**3**). Only one non-hospitalized patient did not develop measurable amounts of D614G virus-neutralizing antibodies (**Figures 2**, **3**). Of note, an increase in the NT/ELISA ratios, and thus the functional quality of the antibodies, occurred in the three groups up to six months, which is most likely a consequence of the affinity maturation of IgG over time, accompanied by the maintenance of high-affinity SARS-CoV-2 neutralizing antibodies and the decay of low-affinity antibodies (28, 29). Continuous somatic hypermutation due to a certain degree of antigenic persistence and ongoing germinal center reactions after infection with SARS-CoV-2 or vaccination were shown to increase the breadth of antibody responses and neutralizing potencies, including higher resistance to mutations in the RBD (29, 30).

We found that the range of specific neutralizing activities after SARS-CoV-2 infection was larger than after COVID-19 vaccination (**Figure 5**; **Supplementary Figure 2**), consistent with other studies (4, 16). One reason for the observed greater heterogeneity could be differences in the S sequences of infecting strains in contrast to vaccinees, who obtain exactly the same construct and had more homogeneous antibody responses. A recent study has indeed shown that samples derived from Beta and Gamma VOC-infected patients are less able to neutralize the Delta VOC than samples from patients infected with early pandemic strains or the Alpha VOC (31). In our work, sequencing of strains before the appearance of VOCs reflected the relatively low genetic diversity of circulating strains in Austria during this time period, with some lineages occurring at higher frequency in the general population (e.g., B.1.160 with S:N477K, B.1.258 with S:N439K, or B.1.177 with S:A222V) (32). Nevertheless, we detected different patterns of specific neutralizing activities in infected patients associated with mutations in the NTD (**Figure 6B**). We observed lower specific neutralizing activities in samples of individuals infected with a mutated NTD strain, which was significant for the Beta VOC, but not the Delta VOC (**Figure 6B**). This could be explained by different NTD mutations present in these two SARS-CoV-2 variants (33), allowing the Beta VOC a stronger immune evasion from antibodies induced by the infecting strains sequenced in this study. Most of the neutralizing activity is directed against the RBD as shown by depletion analyses (11, 16), but strongly neutralizing antibodies recognizing the NTD were also isolated from infected patients (4, 13, 14, 34). Mutations in the NTD can not only directly change antibody-binding sites, but can also have long-range effects on the structure of S, thus reducing antibody recognition (15, 31, 35, 36), which might have contributed to our results. A limitation of our analyses is that we had only a subset of patients with a known S sequence, and we could only evaluate the patterns early after infection. Since we have seen an improvement of the specific neutralizing activity over time (**Figure 5**), it would have been of interest to find out whether such an effect still exists in later samples. Additional factors, like antibody subclass distribution and/or posttranslational modifications of RBD- and/or S-specific antibodies, should be investigated in future studies addressing differences in neutralizing activities of vaccine- and infection-induced antibodies.

Our results also have practical implications. Neutralizing antibodies are important for assessing the quality of immune responses, but NTs are more time-consuming than ELISAs and require the handling of infectious virus. It would thus be of interest to relate ELISA values to NT titers and define a BAU/ml threshold for neutralization, which, however, has to take the source of immunity into account. Even more complex is the relation of these values to protection, as Fc-mediated effector functions of non-neutralizing antibodies can also play a role (37, 38). The relatively small sample size is a limitation of our study. Thus, further studies are required to precisely characterize the different functional properties as well as temporal development of antibody immunity induced by SARS-CoV-2 infections and/or COVID-19 vaccinations in larger cohorts, which can provide leads for the design of next-generation vaccines as well as help in the definition of a generally applicable correlate of protection.

## Data Availability Statement

The datasets presented in this study can be found in online repositories. The names of the repository/repositories and accession number(s) can be found in the article/**Supplementary Material**.

## Ethics Statement

The studies involving human participants were reviewed and approved by the Local ethics committee of the Medical University of Vienna (EK-No. 1291/2021 and 1926/2020). The patients provided their written informed consent to participate in this study.

## Author Contributions

KS, JA and MG: conceptualization and writing. KS, JC, SA: methodology. KS, JA, MG, JC, SA: investigation. JA, MG, E-PS, LW, MT, WH, AZ: resources. JA: funding acquisition. KS and JA: supervision. All authors contributed to the article and approved the submitted version.

## Funding

The study was supported by the Medical-scientific fund of the Mayor of the federal capital Vienna [grant Covid003].

## Conflict of Interest

The authors declare that the research was conducted in the absence of any commercial or financial relationships that could be construed as a potential conflict of interest.

## Publisher’s Note

All claims expressed in this article are solely those of the authors and do not necessarily represent those of their affiliated organizations, or those of the publisher, the editors and the reviewers. Any product that may be evaluated in this article, or claim that may be made by its manufacturer, is not guaranteed or endorsed by the publisher.

## Footnotes

1
https://www.who.int/en/activities/tracking-SARS-CoV-2-variants/
