# Supplementary material for: Heterogeneous SARS-CoV-2-Neutralizing Activities After Infection and Vaccination
Source: Front Immunol. 2022 May 30;13:888794. doi: 10.3389/fimmu.2022.888794 (PMC9196185; doi:10.3389/fimmu.2022.888794)
Supplement: Supplementary file 1 [file DataSheet_1.pdf]

Supplement

Heterogeneous SARS-CoV-2-neutralizing activities after infection and vaccination

Marianne Graninger[1], Jeremy V. Camp[1], Stephan W. Aberle[1], Marianna T. Traugott[2], Wolfgang Hoepler[2], Elisabeth Puchhammer-Stöckl[1], Lukas Weseslindtner[1], Alexander Zoufaly[2], Judith H. Aberle[1]†, Karin Stiasny[1]†*

[1] Center for Virology, Medical University of Vienna, Vienna, Austria

[2] Department of Medicine IV, Clinic Favoriten, Vienna, Austria

† These authors have contributed equally to this work and share last authorship.

*** Correspondence:**
Karin Stiasny
karin.stiasny@meduniwien.ac.at

Keywords: SARS-CoV-2, COVID-19, SARS-CoV-2-specific antibody responses, SARS-CoV-2-neutralizing antibodies, variants of concern

Supplementary Table 1: Reduction in RBD IgG concentrations and neutralization titers from early to late sampling point.

	n/N (%)*	Fold reduction of antibody concentrations (comparison of early and late samples), mean ± SD**			
		**RBD IgG BAU/ml**	**D614G NT titers**	**Beta NT titers**	**Delta NT titers**
**non-hospitalized**	9/24 (37.5)	2.4 ± 1.8	1.8 ± 1.4	1.4 ± 1.2	2.0 ± 2.3
**hospitalized**	22/23 (95.7)	11.5 ± 14.0	4.7 ± 3.9	4.2 ± 4.2	3.9 ± 4.0
**BNT162b2 vaccinated**	26/34 (76.5)	15.5 ± 6.4	4.8 ± 2.9	3.6 ± 2.4	6.9 ± 4.5

* n, number of patients positive at both time points; N, number of patients for which early and late samples were available; (%) percentage of patients positive at both time points.

** Reduction was calculated on an individual basis with patients positive in all assays at both time points.

Supplementary Table 2: Variation of specific neutralizing activities (NT/ELISA ratios)

	Maximum-to-minimum factors of NT/ELISA ratios*	
	"early" samples**	"late" samples**
	**D614G NT**	
**Non-hospitalized**	26.4	41.0
**Hospitalized**	24.6	13.9
**BNT162b2 vaccinated**	9.8	4.4
	**Beta VOC NT**	
**Non-hospitalized**	49.1	18.5
**Hospitalized**	69.1	37.1
**BNT162b2 vaccinated**	7.5	5.6
	**Delta VOC NT**	
**Non-hospitalized**	53.0	19.4
**Hospitalized**	29.6	35.5
**BNT162b2 vaccinated**	12.9	8.1

* Data used for factor calculations are shown in Figure 5 (D614G NT/ELISA ratio) and Supplementary Figure 2 (Beta VOC NT/ELISA ratio, Delta VOC NT/ELISA ratio). Maximum-to-minimum factors were obtained by dividing the maximum ratio through the minimum ratio.

** Early samples were taken approximately 3 weeks (median) and late samples approximately 6 months (median) after infection or 2nd vaccination (see Table 1).

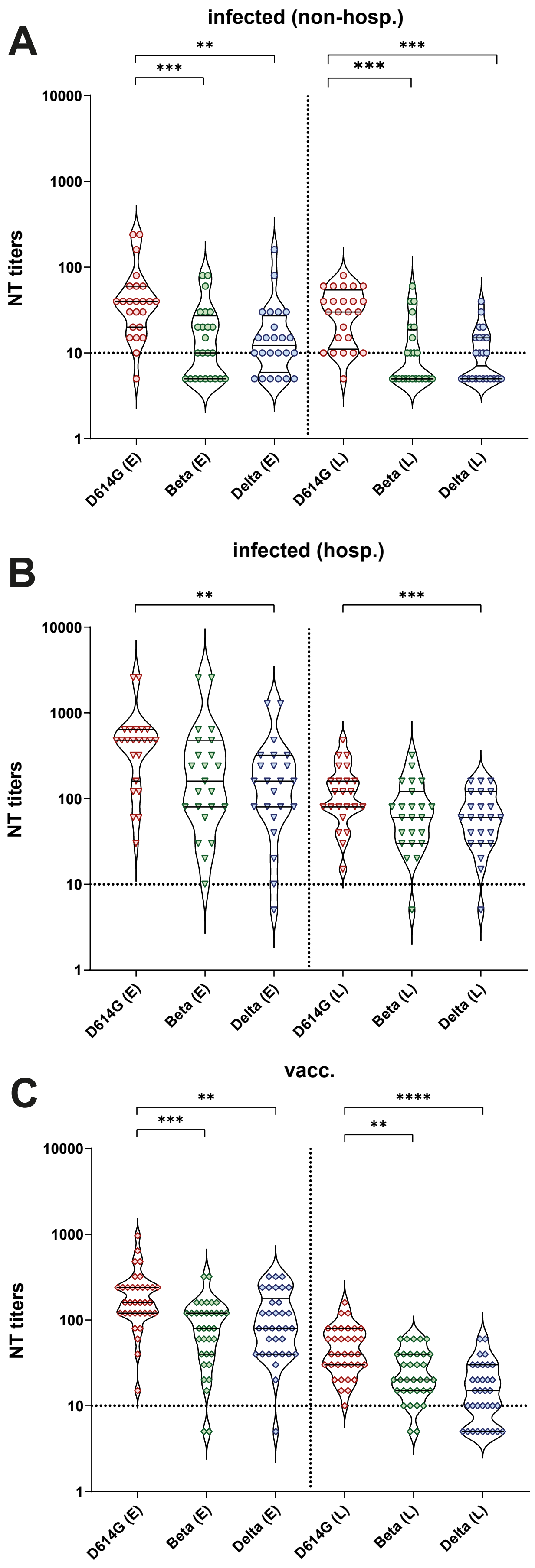

A
infected (non-hosp.)
**
***
***
***
NT titers
10000
1000
100
10
1
D614G (E)
Beta (E)
Delta (E)
D614G (L)
Beta (L)
Delta (L)
B
infected (hosp.)
**
***
C
vacc.
**
***
****
**

Supplementary Figure 1: Neutralizing antibody titers against an ancestral D614G virus strain and the two VOCs Beta and Delta after infection (non-hospitalized and hospitalized patients) and two doses of the mRNA vaccine BNT162b2, compared between SARS-CoV.2 variants within cohorts. (A) Non-hospitalized patients, (B) hospitalized patients, (C) vaccinees. Early samples (E) were taken approximately three weeks after disease onset or the second vaccine dose; late samples (L) approximately six months after disease onset or the second vaccine dose. Bars within the violin plots indicate the median with interquartile range, the dotted horizontal line the cut-off of the assay. Statistical analysis was performed with the Kruskal-Wallis test and Dunn's multiple comparisons *post hoc* test. Asterisks indicate statistical significance: (*) = $P \leq 0.05$, (**) = $P \leq 0.01$, (***) = $P \leq 0.001$, (****) = $P \leq 0.0001$. non-hosp., non-hospitalized patients; hosp., hospitalized patients; vacc., vaccinees.

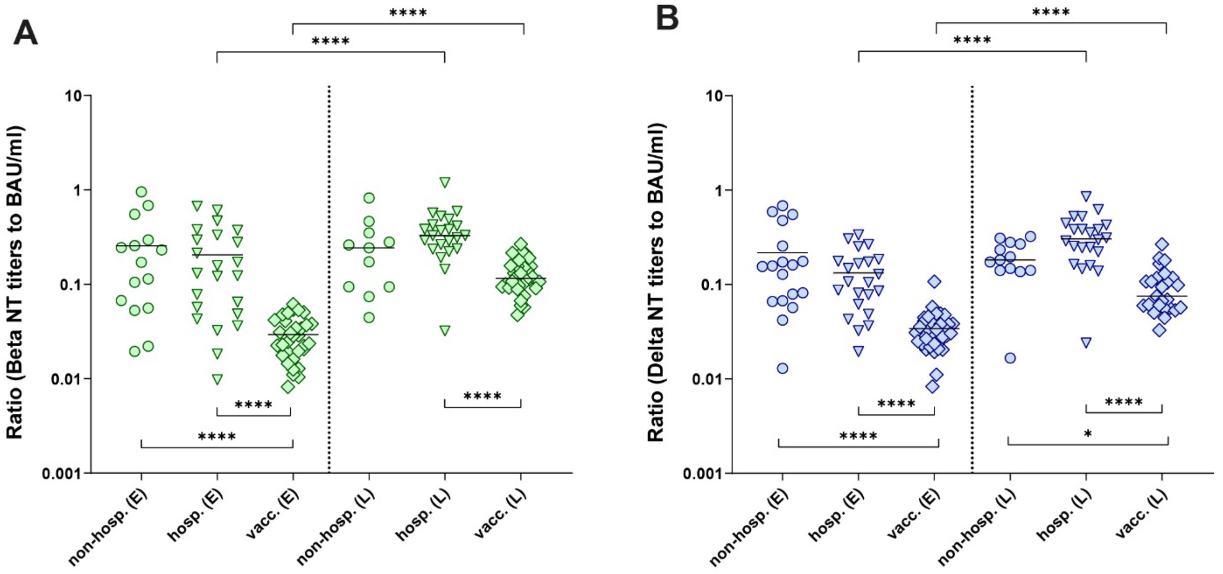

Supplementary Figure 2: Ratios of VOC NT titers and ELISA concentrations of individual serum samples after infection (non-hospitalized and hospitalized patients) and two doses of the mRNA vaccine BNT162b2. (A) Beta VOC, (B) Delta VOC. Only NT- and ELISA-positive samples were included. Early samples (E) were taken approximately three weeks after disease onset or the second vaccine dose; late samples (L) approximately six months after disease onset or the second vaccine dose. Bars indicate the mean. Statistical analysis was performed with ANOVA and Tukey's multiple comparison's *post hoc* test (comparison of cohorts, bottom brackets) and paired t-test (comparison within groups, top brackets). Asterisks indicate statistical significance: (*) = $P \leq 0.05$, (**) = $P \leq 0.01$, (***) = $P \leq 0.001$, (****) = $P \leq 0.0001$. non-hosp., non-hospitalized patients; hosp., hospitalized patients; vacc., vaccinees.

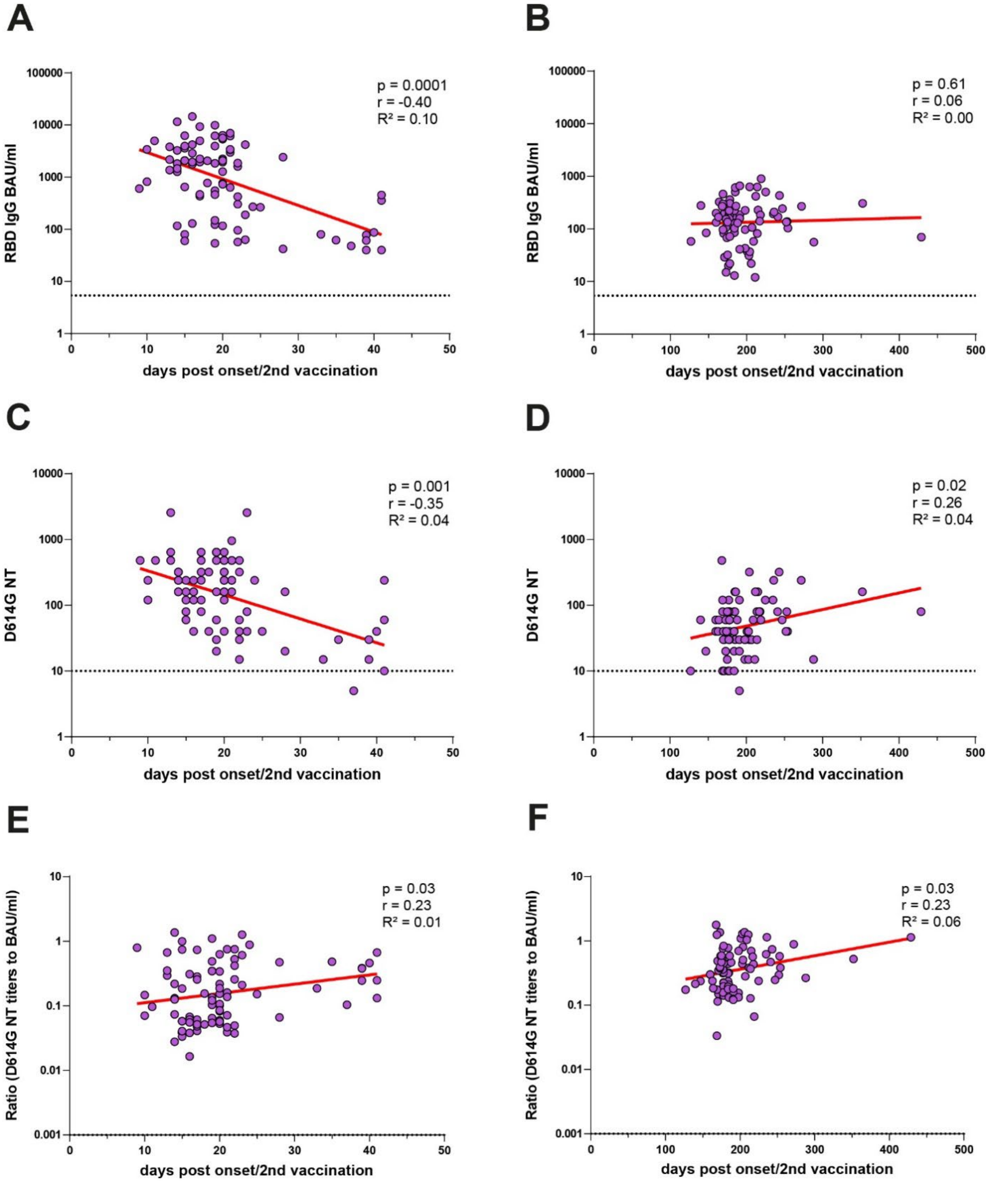

Supplementary Figure 3: Correlation analyses of D614G-specific antibody metrics of all samples plotted against days post onset or 2nd vaccination. Spearman correlation coefficients (r) and coefficients of determination (R^2) are indicated. Linear regression lines are shown in red. (A-B) IgG BAU/ml versus early and late sampling time points, respectively; (C-D) D614G NT titers versus early and late sampling time points, respectively; (E-F) NT/ELISA ratios versus early and late sampling time points, respectively. As in Figure 5, for assessment of ratios samples from infected and vaccinated individuals for which early and late samples with a positive result in NT and ELISA were included. Only one non-hospitalized patient had to be excluded because of a negative NT result.
